# Pyorrhœa Alveolaris

**Published:** 1904-04

**Authors:** B. F. Arrington

**Affiliations:** Goldsboro, N. C.


### PYORRHŒA ALVEOLARIS.

BY DR. B. F. ARRINGTON, GOLDSBORO, N. C.

THIS may to many seem to be almost a threadbare subject, but it is not, and it must and will be discussed in the interest of humanity until a reasonable, practical, and definite line of treatment that will cure shall be established and accepted by the profession, and in dental colleges proclaimed to students as orthodox and reliable, and students shall be taught that the disease, when uncomplicated, is amenable to a simple, practical line of treatment, and is speedily curable, and that there is no need for all the *big noise* some are making about extreme features of the disease and difficulty in effecting cure.

I have for several years past been impressed with the idea that some men in the profession when writing about and discussing this very prevalent disease, especially some of those professing to make a specialty of the treatment, were in effort making a mountain of a very small hill. Upon reflection, since attending the meeting of the National Dental Association at Asheville, and more recently reading a most ably written, but extravagant paper on the subject, by a prominent dentist of Chicago, and published in the *Dental Summary,* June and July issues, 1903, I am more forcibly impressed with the fact, and can plainly see, as many do, that there has been much buncombe indulged by some, with a very great amount of unnecessary extravagant talk about the disease, and largely magnified difficulty of treatment. The extreme features in practice so conspicuously proclaimed and indulged by that class of practitioners, singly or as a whole, have not evidenced any marked ability as practical practitioners, especially as pertains to this disease.

During the meeting of the National some very extravagant and out of place "spread-eagle" utterances were indulged in in relation to treatment of the disease by dentists who were seemingly disposed to impress listeners with the idea that they certainly knew it all, and held the subject definitely in hand, and could dispense treatment successfully beyond a question of doubt, and that the disease was something wonderful to treat, and was the disease of all diseases difficult to treat, and that so and so, especially the use of sharp scalers in the hands of experts, were requisites for success.

One dentist of some prominence, much heard, ventured to say that he did not know to what to attribute his success in the removal of deposits and his successful treatment of the disease, unless it was his peculiar sensitiveness of touch in the use of scalers; and similar expressions, evidently designed to convey the impression that treatment was extremely difficult, and but few men were equal to the task. Others talked on the same line with marked extravagance of expression, and as much out of place considering the occasion, but were not quite so flippant and harmful in effect. Some have boasted of their high charges for treating the disease, mentioning bills rendered and paid, for hundreds of dollars, thousands, for treating a single case. Unquestionably feeling (false assumption) that they were moving on a high plane, as was the case (pitiable) with some men in the profession several decades back, who were addicted to boasting on public occasions of their high charges, fifty or a hundred dollars for a single gold filling. Not much to their credit in the face of the fact that there were numbers of dentists all around them that could and were probably inserting fillings, daily, as perfect, possibly, for less than one-tenth the amount. But so it is, and long has been, a weak, corrupting, and demoralizing feature in practice, but possibly, very probably, will continue to prevail until the profession in open meeting condemns such unprofessional conduct, and demands clean expression, clean action, and no trick and trade or humbuggery in dental practice, and put a stop to the existing crying shame that is so unbecoming and hurtful to the status and dignity of dentistry.

Some harangues at the National meeting were listened to with considerable feeling of disgust by many present, and with much desire and hope that a different order of things may soon prevail, and the National will be national truly, in work, dignity, and elevation. Some men of distinguished prominence in the profession are accountable, to a large extent, for much of the prevailing extreme and unreasonable opinions now entertained concerning pyorrhœa alveolaris as a disease, and the want of an established, correct line of treatment.

Many have written and talked entertainingly on the subject, but few wide of the mark for successful treatment. I will, in connection with the paper in the *Summary* above mentioned, enumerate some of the extravagant sayings and false teachings on the subject that have tended to confuse and mislead rather than enlighten and establish correct views and a curative line of treatment.

A recent writer has said, "This disease is peculiar, in that it is often found in mouths the care of which has been rigidly looked after. . . . As a rule, if the pulp is removed at the first evidence of developing pyorrhœa, the disease is arrested." He mentions the use of solvents, and advises the "use of cauterants of such strength that it is requisite to protect the mouth; also to use a soft rubber brush after treatment." All of which evidences plainly his utter ignorance of the disease uncomplicated, and his inability to treat successfully.

A dentist of decided ability, and regarded by those competent to measure professional merit as a first class practitioner, has said, in discussing a paper pertaining to treatment of pyorrhœa, "that in one instance, after several years treatment of a case and failure to cure, he cut off the natural crown and substituted an artificial crown, and cure followed." Truly wonderful! why should he have conceived such an idea? No explanation offered, and it is presumable that none could be given. Another dentist in Chicago, quite as prominent, has said,—

"I do not remove deposits at first sitting, but obtain a sample of patient's urine and examine it, and immediately request patient to drink from eight to twelve glasses of water daily." He further advises to "lance the gums if much inflamed," and he ventures the utterance that "modern dentistry is producing more pyorrhœa than any other cause." And then, to cap the climax and make absurd more absurd, he says, "Since nature tolerates lime deposits in all parts of the body, it would seem immaterial whether the deposits are removed from the roots or not. . . . Nature takes care of all such deposits." He also advises, "in place of instruments for removal of deposits, the use of a dissolving fluid, especially when the disease is extensive." He does not name the fluid, possibly an unfortunate oversight that may yet be remedied, but I think it questionable.

Some dentists, embracing some of the ablest men in the profession, contend that "extraction is the only means of cure." Strong men on some lines, doubtless, but as pertains to treatment of pyorrhœa, weak theory and condemnable practice. Others advise to "sink bars in the crowns, and to drill holes in the teeth when loose and ligature to hold firm." And another has said,

"Treat with cautery and burn it out as you would a fistula." Some say "the disease is confined exclusively to middle life, and is always found in connection with good teeth, exempt from decay." Truly a beautiful display of ignorance concerning the disease. Such is inexcusable for dentists of experience and prominence, but so it is, and there is much of it.

One dentist, quite notable, advises "that patients shall smear the ends of two or three fingers with vaseline and menthol and rub the gums surrounding all the teeth for five minutes twice daily." Presumably, very large mouths and small fingers. Whether this treatment is for several days only, or during life, is not stated.

Some contend that when the alveolar process has wasted half or two-thirds, exposing roots of teeth, with gums festooned and flapped to the depth of alveolar waste, they can, by a certain (not explained) line of treatment, "restore the process to original proportions to necks of teeth, and establish normal attachment of gum tissue to the cementum." If such were possible, what cause for rejoicing! but it is not possible. There is no reason why any one shall be deceived about such a result. To carefully watch the treatment is all that is requisite to establish facts that will be convincing. Another dentist somewhat prominent, but very impractical and sensational, says, "When teeth are very loose, the first consideration shall be the devitalization of the pulps." Most absurd and very misleading. Then comes another, quite his equal in radical extreme, in treatment, one who has written and talked much about the disease, and says, "Treatment consists in patient's drinking eight or nine glasses of water each day, in brushing the gums with a stiff brush to make them bleed, and the employment of proper mouth-washes." A shot far from the mark for one so notably prominent as a teacher, and a treater of this disease, but nothing but irrational or extreme on this line need be surprising, since so many of much prominence, and some of little prominence, have so wildly side-tracked and drifted along with a wild craze on the subject, and are making guess-work the foundation basis of theory and practice.

Some advise to "lay open the gum over each tooth, and to cauterize the surface of the flaps with nitrate of silver and carbolic acid." Then follows advice of others to "cut away the lateral flaps and apply the hot iron (galvano-cautery) over the cut surface of all the affected teeth in one or several days." Others say,

"Scrape away the cementum, also the alveolar process, and to get at it successfully cut through the gum, then close with sutures."

Can anything in line of treatment be more unreasonable, the nature of the disease considered? But so it is. Some one anxious for notoriety will bleat out something as a new feature in treatment, whether in accord with reason and sound judgment or no, and the bleating is taken up by apists, and so goes, passing down the line with loud acclaim based on ignorance. A conspicuous dentist of California, rather sensational and not very practical, advises to "pass silk thread through the gums, both external and internal spaces on each side of the teeth, and to ligate the gums in contact with the teeth."

For what purpose not stated, and no one can imagine. Yet he has his followers to endorse and herald such a feature in practice as requisite and important, and the craze goes on.

Dr. M. has recommended "cauterization of the pockets with pure chromic acid, the use of chlorate of potash, scarification of the gums, leeches, and purgative mineral water." Another of much reputation suggests "partial destruction of the gum forming the alveolar pockets, followed with bichloride solution." Another, in New York, of considerable notoriety, but quite as impractical for successful treatment as the distinguished Californian, has advised "to fill the pockets with paste composed of two-thirds caustic potash and one-third crystallized carbolic acid." Pretty severe treatment for any disease short of cancer; but some will argue that it is from a New York dentist of note, and must be good practice.

Others advise the use of "pure sulphuric acid, full strength, to burn out pockets and decalcify the cementum." This is heroic out of reason, and must do more harm than good.

The "wearing of copper plates and bands for a year, and to be continued longer if necessary," was advised and practised by a New York dentist, one of the most notable and conspicuous men that have ever joined the ranks of the dental profession, but no favorable report was ever made of such coppery treatment, and none could reasonably be expected.

A dentist now prominent in New York insists "that pyorrhœa alveolaris is always caused by syphilis, and he treats accordingly. Such may be the lamentable condition of New York patients, but it is questionable, and when so treated we can easily guess the result. Now comes a fancy delicate feature in dental surgery that almost tempts a smile. Dr. H., of Chicago, advises, when there is much receding of the gum, to "make an incision in the gum a little below the border and insert crystals of iodide of zinc; this irritates the gum, pushing it up on the tooth where it adheres." When the doctor can produce positive evidence of *such adhesion,* then will be time to credit and sanction such practice, not before. One small practical fact is of more intrinsic value in practice than a thousand fine-spun fancy theories. Give us facts foundationed upon results obtained; that is what we want.

Some are recorded as advocates of "lactic, trichloracetic, and sulphuric acids as solvents of pyorrhœa deposits," and profess to obtain satisfactory results. Such is deception. The deposits cannot be dissolved or even softened in the mouth by any acid, liquid, paste, or powder known to dentists or chemists without serious detriment to soft tissues. Some advise "hot baths, cold baths, Turkish baths, life on sea-shore, sea-bathing, resort to mountain sections, open-air exercise, and heroic exercise as essential for cure of the disease."

If this be true, there is but little chance for cure of the poor as a class, who are most afflicted with the disease. They are ignored entirely. Such treatment may be admissible and may help to cure sometimes, but certainly it is, to say the least, entirely out of the reach of the masses. Others contend that "perfect occlusion is requisite for successful treatment," and they shorten teeth and rearrange accordingly, and then fail of desired results.

One Chicago dentist advises: "If a three months' course of treatment does not affect a cure, let the patient go for a month; then give a second trial of same vigorous treatment for another three months." Most unreasonable. A true case of pyorrhœa alveolaris (Riggs's disease), or *interstitial gingivitis,* if disposed to so call it, a true typical case, uncomplicated, can be as successfully treated in from three to four weeks as in three months or three years. The thing requisite is to do the work thoroughly in removal of deposits, and treat mildly.

Dr. P., of Philadelphia, a superior man and a splendid dentist, has said, "The disease commences at the apex of the roots," and he sticks to it, right or wrong, in the face of much convincing argument and evidence to the contrary. Such, however, is human nature, and now and then will crop out and display conspicuously, whether for good or evil.

Most dentists who have written about and discussed treatment of this disease have advocated use of sharp scalers, and herein lies the failure of successful treatment. Some say, "Curette the cementum, and cut or scrape away the edge of the alveolar process." Possibly, but few greater blunders pertaining to treatment of the disease have ever been perpetrated.

Now we approach the zenith of all treatment that has ever been proposed, possibly the most unreasonable, extreme, and unjustifiable as a whole, that ever has or ever will be perpetrated in civilized communities. The doctor in question writes considerably, and most extravagantly, on the subject. Too much of it to relate in full; it would be overtaxing. I will quote only a brief clipping from a dental journal. Just a little of such will suffice to enlighten or disgust. He says, "Rinse the mouth with permanganate of potash solution, one-half grain to ounce of water. Touch the gums with carbolic acid, and inject five per cent. solution eucaine; then carry down quickly, from gum margin to alveolar process, a three-edged flexible lancet, passing it around the teeth, severing gum entirely from teeth. With scraper or chisel scrape away the diseased pericementum, the external layer of the cementum, and the diseased portion of the process. Success depends upon the thoroughness with which this is done, and it requires skill and practice. Wash out the pocket, and with cotton wrapped on a broach protect the mouth and carry to the bottom of the pocket sulphuric acid full strength. Wipe away oozing blood, and repeat, holding the acid in contact until the root surface is decalcified. Rinse mouth with soda solution, and prescribe as mouth-wash permanganate of potash one-quarter grain to ounce of water, used hourly until gum heals. A dose of Epsom salt daily for three weeks, also sarsaparilla and potassium iodide three times a day. Devitalize the pulp in all cases presenting in advanced stages. In very advanced cases cut off crowns just above gum level. After root treatment, crown, and solder crowns together if several."

If this treatment fails to cure, most probable; but should it kill, who would be surprised?

In my humble opinon, just so long as dental journals will consent to accept and publish papers taxing the profession with so much of such conglomerate, impractical treatment as above mentioned, we cannot reasonably hope that the disease will be better comprehended or more successfully treated than at present. It is but seldom we read a paper pertaining to the disease that is reasonable, conservative, and practical. Hence the much leading astray that has prevailed.

Pyorrhœa alveolaris is a very common disease, possibly of more frequent occurrence, in many sections of country, than any disease known to man. It is easily recognized, and can be easily treated, with good results following if rightly treated on a plain practical basis of local procedure, provided there is not syphilitic complication, as specified by a New York dentist. In treatment of the disease there can be no justification for severe or prolonged treatment, nor for excessive charges for treatment. Maltreatment and overcharging for same is censurable and condemnable.

The thing requisite for success in treatment is the *thorough removal of deposits* from the roots of teeth, thorough as thorough can be practically, no guesswork about it. This accomplished, the requisite following treatment of soft tissues is simple and easy, and cure speedily follows in a large percentage of cases.

In the removal of deposits it is all important to avoid, as far as possible, injury of the soft tissues, the cementum, and alveolar process. Hence the necessity for use of *smooth-edge scalers.*

The time limit for cure of a typical case, pus discharging and teeth loose in sockets, does not exceed from three to four weeks, seldom more than three, and a large majority of cases presenting for consultation or treatment can be successfully treated in less time than two weeks.

One sitting for removal of deposits is all that is requisite in most cases, and is best for patients. The removal of deposits with smooth scalers is much more expeditious than with sharp ones. But few remedies (two or three), campho-phenique chiefly, for the healing of gums and restoration to a healthy state, will accomplish as much or more than dozens, as advised by some.

The use of toothpick and tooth-bursh after meals daily, after the removal of deposits and treatment, is important for effective cure and to prevent return of the disease. Patients must follow instructions as to use of tooth-brush if they wish to preserve and enjoy the blessing of a healthy mouth, teeth and gums especially.

I hold that any member of the profession can do, in the treatment of this disease and the accomplishment of results, just what any other member can, accepting the fact that the disease is local and is curable, and he will not try to do too much after successful removal of deposits, and will discard from mind all thought of impractical papers and discussions, and false teaching on the subject.

From experience in practice, and watchful observation of results following treatment, I feel justified in saying that any practical dentist of fair comprehension, can, in a few hours' observation of the use of smooth scalers in the removal of deposits, be able to manipulate such instruments effectively, and will fully realize in a limited period of time that the deposits can be removed more quickly and more thoroughly than with sharp scalers, and will feel satisfied that successful treatment of the disease is possible with the whole profession, if desired, and all can treat and cure if they will. And that all this extravagant sensational talk and writing about the disease, and the difficulty of treatment and cure, is unnecessary and unauthorized, and cannot be sustained by facts demonstrated through a correct line of practice or otherwise.

Shall sensational writing be longer indulged? Is there need for it? Can good come of it? are questions that present themselves for thoughtful consideration.

Let us work on right lines for good results, that the public may be more favorably impressed and definitely realize the fact that we can, as a whole, treat successfully for relief. The idea that a disease of so small compass, never extending to greater extent than the length of the roots of teeth, so easily reached and so easily treated, should be such a stumbling block to the profession as it has been, and baffled the best skill in dental ranks for these many years, half a century or more, does not speak well for the advance of the profession, and it is surprising and humiliating. It is not that the disease is so difficult to treat that has caused failure of general success in treatment, but the much false theory and teaching as to etiology and treatment that has been heralded through papers and discussions on the subject.

May we not reasonably hope that the day is not far distant when the profession will know more about this disease and better how to treat it than at present, more humanely and practical, requiring less time and fewer remedies with better results?

In conclusion, I feel that I cannot do better for the profession and the public than to advise old and young practitioners to procure if possible, and read carefully, a paper, "Pyorrhœa Alveolaris," written by Dr. Henri Letord, of Orlando, Fla., published in

*Dental Hints,* May issue, 1903, which, as a whole, so far as it goes, is the best paper I have ever read pertaining to this disease. Practice based upon such a foundation of theory as he has laid down could but promise good results, and would do away completely with all this big craze so long indulged concerning cause and treatment of the disease.

When the principles of prophylaxis shall be more generally understood, and shall be taught in dental colleges (not on extreme lines), and the practice of prophylactic treatment for purification and health of mouths shall be more universally indulged than at present, there will, proportionately, be less of pyorrhœa alveolaris to treat, and less of tooth decay and other abnormal features in the mouth, and there would be unquestionably a better state of general health.

The possibility of satisfactory results will justify the expenditure of time, trouble, and expense requisite in experimenting for truth and conviction that will be realized through watchful observation of results.

